# Comparative efficacy of fruquintinib, regorafenib or TAS-102 in patients with KRAS-mutant metastatic colorectal cancer: a systematic review and network meta-analysis

**DOI:** 10.3389/fonc.2026.1948508

**Published:** 2026-10-16

**Authors:** Yingyu Zhou, Shiqing Zhao, Xiaoyi Zheng, Yixuan Wang, Mingzhen Wang, Siyu Chen, Zhenan Wu, Dong Zang, Wenzhe Si

**Affiliations:** 1 Department of Laboratory Medicine, Beijing Hospital of Integrated Traditional Chinese and Western Medicine, Hospital of Integrated Traditional Chinese and Western Medicine, Beijing University of Chinese Medicine, Beijing, China; 2 Department of Laboratory Medicine, National Health Commission (NHC) Key Laboratory of Cardiovascular Molecular Biology and Regulatory Peptides of National Health Commission, State Key Laboratory of Vascular Homeostasis and Remodeling, Peking University Third Hospital, Beijing, China; 3 Department of Biology, The College of Life Sciences and Health, Wuhan University of Science and Technology, Wuhan, Hubei, China

**Keywords:** fruquintinib, KRAS, metastatic colorectal cancer, regorafenib, TAS-102

## Abstract

**Objective:**

This study aimed to synthesize evidence from recent randomized controlled trials (RCTs) evaluating the efficacy of regorafenib, fruquintinib, and TAS-102 in patients with metastatic colorectal cancer (mCRC). Using a network meta-analysis, we compared long-term survival outcomes (overall and progression-free survival) in the overall population and in patients with KRAS mutations, to guide treatment decisions and inform therapy selection in molecularly defined subgroups.

**Methods:**

We systematically searched PubMed, Embase, and the Cochrane Library up to 6 November 2025 for randomized controlled trials reporting overall survival (OS) and/or progression-free survival (PFS) in patients with metastatic colorectal cancer (mCRC). Risk of bias was assessed with the Cochrane RoB-2 tool and heterogeneity with I2. A Bayesian network meta-analysis using non-informative normal priors and a random-effects consistency model was fitted; convergence was assessed with the Gelman-Rubin diagnostic. Comparative safety was not formally meta-analyzed.

**Results:**

Ten RCTs (n = 3959) were included, with no randomized head-to-head comparisons among the active treatments. Compared with placebo, all active treatments significantly improved overall survival (OS): fruquintinib HR 0.66 (95% CrI 0.53-0.84), regorafenib HR 0.69 (95% CrI 0.51-0.88), TAS-102 HR 0.69 (95% CrI 0.55-0.85), and TAS-102 plus bevacizumab HR 0.41 (95% CrI 0.28-0.59). Combination therapy ranked first; indirect differences among the three single agents were not consistently statistically significant. In patients with KRAS-mutant tumors, fruquintinib and TAS-102 were associated with numerically favorable OS estimates compared with placebo [HR 0.71 (95% CrI 0.51-1.01) and 0.74 (95% CrI 0.50-0.99), respectively], whereas regorafenib did not [HR 0.81 (95% CrI 0.52-1.19)]. These statistical differences versus placebo do not demonstrate superiority over regorafenib, and rank probabilities are exploratory.

**Conclusions:**

Fruquintinib and TAS-102 plus bevacizumab are effective later-line options for mCRC. In patients with KRAS-mutant CRC, fruquintinib may be a reasonable option, but available indirect evidence does not establish its superiority over TAS-102 or regorafenib. These findings provide a trial-level, KRAS-stratified synthesis of the available placebo-controlled RCT evidence and should be interpreted as hypothesis-generating, rather than as evidence of a predictive effect of KRAS status or of rank-based superiority among the agents.

**Systematic Review Registration:**

https://www.crd.york.ac.uk/prospero/display_record.php?ID=CRD420251275994, identifier CRD420251275994.

## 1 Introduction

Colorectal cancer (CRC) is the third most common diagnosed malignancy worldwide and the second leading cause of cancer-related death. Patients with stage IV CRC have a poor prognosis, with a 5-year survival rate of only 14% (1). Studies show that mutations in the RAS gene family play a key role in driving CRC progression and have substantial clinical relevance (2). The RAS protein family relays signals that control cell growth, division, and survival (3). Among its three main members KRAS, NRAS, and HRAS, KRAS is the isoform most frequently altered by point mutations, accounting for approximately 85% of all RAS alterations in CRC, while NRAS and HRAS are affected in only 12% and 3% of cases, respectively (4). Overall, about one-third of CRC cases harbor a KRAS mutation, with the single-base substitution leading to the G12D variant being the most common (5). These mutations reduce the efficacy of several targeted therapies. Five principal mechanisms explain most of this resistance: (1) amino-acid substitutions such as R68S, Y96C, or Y96D reshape the drug-binding pocket and weaken inhibitor contact; (2) in KRAS G12C-mutant cells, increased protein expression shifts the equilibrium toward the active, GTP-bound state, which current inhibitors cannot effectively target; (3) feedback reactivation of upstream receptors such as EGFR can re-engage the RAS pathway even when the mutant protein is blocked; (4) persistent activation of focal adhesion kinase (FAK) following G12C inhibition disrupts the FAK–YAP axis and promotes fibrosis, ultimately constraining drug efficacy; and (5) epithelial-to-mesenchymal transition (EMT) reprograms receptor expression upregulating ERBB3 or FGFR1 and thereby boosts parallel PI3K/AKT and MAPK signaling pathways that bypasses RAS blockade (3).

Since the discovery of the RAS gene family in 1980, more than three decades have been spent searching for drugs capable of inhibiting its activity. The effort has ultimately yielded agents such as sotorasib (AMG-510) and adagrasib (MRTX849), which specifically target the KRAS G12C mutant and have shown clear benefit in CRC. Unfortunately, tumors harboring other KRAS alterations still lack corresponding targeted therapy; for these patients, standard care continues to rely on broad-spectrum kinase inhibitors and cytotoxic chemotherapy (6–9).

The 2024 NCCN guidelines therefore recommend RAS and BRAF testing for every patient with mCRC. Cetuximab or panitumumab should not be used either as monotherapy or in combination, in the presence of any activating KRAS or NRAS mutation. The only exception is the KRAS G12C subset, in which the addition of adagrasib to either antibody is encouraged. For all other RAS- or BRAF-mutant cases, fruquintinib is listed as the preferred option (10, 11). Because effective and well-tolerated treatments remain limited for this population, the three oral drugs most commonly used in the third-line setting regorafenib, fruquintinib, and TAS-102 have each been shown to prolong survival and maintain quality of life. These three drugs exhibit distinct mechanisms of action.

Regorafenib (BAY 73-4506, Stivarga) is an oral diphenylurea-derived multi-kinase inhibitor that targets pathways involved in angiogenesis (VEGFR-1, -2, -3 and TIE-2), the tumor micro-environment (PDGFR-β, FGFR), and cell proliferation (KIT, RET, BRAF) (12). It was the first small-molecule kinase inhibitor to improve survival in progressive gastrointestinal stromal tumors, leading to FDA approval in 2012 and 2013 (13–15).

Fruquintinib is an orally available tyrosine-kinase inhibitor that potently inhibits VEGFR-1, VEGFR-2, and VEGFR-3, the main receptors driving tumor angiogenesis (16–18). VEGFR-1 is found on endothelial cells, myeloid monocytes and tumor cells. When ligands such as VEGF-A, VEGF-B or PlGF bind, the receptor promotes angiogenesis by directing inflammatory cells toward the tumor, recruiting bone-marrow progenitors and releasing cytokines that accelerate disease progression (19). VEGFR-2 has the highest affinity for VEGF-A and, despite its weaker binding to VEGF-C and -D, remains the main driver of vascular signaling. Through the PI3K-Akt, MAPK and calcium pathways, it supports endothelial growth, differentiation and migration while blocking apoptosis, thus allowing tumors to survive in hypoxic conditions and adopt a more aggressive phenotype (20, 21). VEGFR-3 is located chiefly on lymphatic endothelium but can also be present on tumor cells. Engagement by VEGF-C or -D promotes lymphatic endothelial proliferation and survival, increasing the density of peri-tumor lymphatic vessels and providing a conduit for cancer cell dissemination via the lymphatic system. In addition, fruquintinib produces modest inhibition of RET, FGFR-1 and c-KIT, although these off-target effects are thought to contribute little to its clinical activity (22).

TAS-102 is an oral fixed-dose combination of trifluridine (FTD) and tipiracil (TPI) in a 2:1 molar ratio (23). FTD, a thymidine analog, acts through two mechanisms. First, it directly inhibits thymidylate synthase, the enzyme responsible for generating thymidine monophosphate required for DNA synthesis during S phase; depletion of this nucleotide halts DNA replication and suppresses tumor-cell proliferation. Second, the FTD metabolite 5-trifluoromethyl-2,4-dioxopyrimidine (FTY) is incorporated into double-stranded DNA, causing miscoding and eventual DNA dysfunction. TPI, a thymidine-phosphorylase inhibitor, prevents rapid degradation of FTD in the liver and intestine, prolonging its half-life and maintaining plasma levels (23–25).

Most efficacy assessments of these three agents have pooled RAS-mutant and wild-type patients without molecular stratification, potentially masking the true treatment effects in specific subgroups and creating a “blind selection” dilemma for clinicians. Furthermore, controversy persists regarding the efficacy of these drugs across different RAS molecular subgroups, introducing uncertainty into precision treatment decision-making (26, 27). Although numerous previous network meta-analyses (NMAs) have compared these three agents, KRAS stratification has not been specifically addressed (28).

Current third-line treatment decisions rely on separate trials that compared each agent with placebo, including the CORRECT and CONCUR trials for regorafenib (29, 30), the FRESCO and FRESCO-2 trials for fruquintinib (31, 32), and the RECOURSE and TERRA trials for TAS-102 (33, 34). This “placebo-controlled” rather than “active-comparator” design has resulted in substantial evidence fragmentation. Most existing meta-analyses have focused on the overall population, while survival data (OS/PFS) specifically examining the RAS-mutant subgroup remain scarce, leading to a lack of evidence-based guidance for treatment sequencing in these patients.

To address these challenges, NMA represents a necessary and appropriate methodological approach. In the absence of direct head-to-head trials, NMA can construct a treatment network using a common comparator (placebo or best supportive care), enabling indirect comparisons across trials. This allows for the estimation of hazard ratios for comparisons such as fruquintinib versus TAS-102 or regorafenib versus fruquintinib, which cannot be achieved within traditional analytical frameworks. Furthermore, NMA provides rank probabilities and SUCRA (surface under the cumulative ranking curve) values, offering clinicians a Bayesian-based approach to ranking treatment options. Therefore, conducting a network meta-analysis comparing regorafenib, fruquintinib, and TAS-102 specifically in the KRAS-mutant subgroup is warranted.

Regorafenib, fruquintinib, and TAS-102 are all established third-line options for mCRC. By pooling recent randomized controlled trial evidence, we aimed to estimate OS and PFS hazard ratios for each drug and to generate hypotheses about the optimal regimen for patients with RAS-mutant CRC, while interpreting treatment rankings as exploratory rather than as evidence of superiority.

## 2 Materials and methods

The research was conducted following the Preferred Reporting Items for Systematic Reviews and Meta-Analyses (PRISMA) 2020 statement (35) and the PRISMA-NMA extension guideline. The protocol was prospectively registered in PROSPERO (CRD420251275994); the KRAS subgroup analysis and the Bayesian network meta-analysis were prespecified as exploratory analyses, and no material deviations from the registered protocol occurred. We searched PubMed, Embase and the Cochrane Library up to 6 November 2025. The initial search yielded 1,180 records. After removing duplicates, studies of non-colorectal cancers and reports that lacked survival data, ten high-quality randomized controlled trials remained; the selection process is outlined in **Figure 1**. (Pubmed: Taking the PubMed database as an example, the standard Medical Subject Heading (MeSH) term “Colorectal Neoplasms” was used. The search also incorporated the drug names Regorafenib, Stivarga, Fruquintinib, Elunate and TAS-102, together with keywords related to patient survival and prognosis).

**Figure 1:**
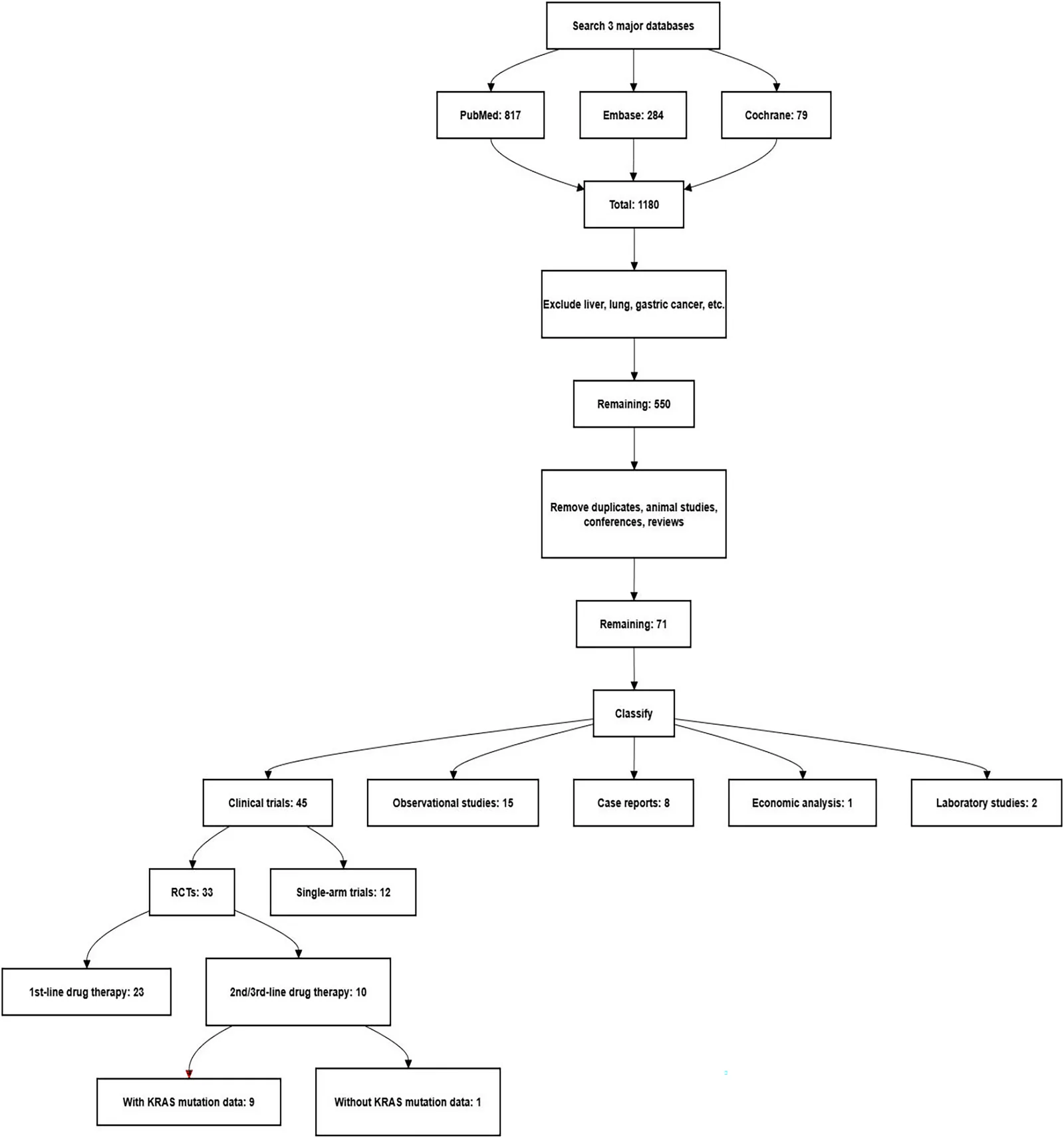
Literature search process.

### 2.1 Study selection and data extraction

Inclusion criteria: (1) Included randomized controlled trial; (2) Adult patients with mCRC who have previously failed second-line therapy and received third-line treatment. (3) Evaluated oral regorafenib 160 mg daily, fruquintinib 5 mg daily, TAS-102–35 mg/m^2^ twice daily (alone or combined with bevacizumab 5 mg/kg), compared with placebo or best supportive care; (4) Reported hazard ratios (HRs) with 95% confidence intervals (CIs) for OS and/or PFS; (5) Provided KRAS-subgroup survival data with corresponding HRs.

Records were screened and data were independently extracted by two reviewers; disagreements were resolved by discussion or referral to a third reviewer. All extracted data were entered into an Excel file. The risk of bias for each trial was assessed with the Cochrane RoB-2 tool. Comparative safety was not formally meta-analyzed because safety outcomes, definitions, and reporting thresholds differed substantially across the included trials; safety information is therefore considered only qualitatively.

### 2.2 Statistical analysis

A Bayesian network meta-analysis (NMA) was conducted on the pooled data using a random-effects consistency model with a common between-study variance. Because the available trials formed a star-shaped network anchored by placebo and had no closed loops among active treatments, formal inconsistency assessment was not possible; consistency was instead supported by the transitivity assessment described below. Hazard ratios (HRs) for OS and PFS, along with their 95% confidence intervals (CIs), were extracted from each study, and the lower and upper bounds of the 95% CI were calculated for subsequent modeling (see formula). (se(log(HR) is the standard error of the log hazard ratio. ln(HRuci) is the natural logarithm of the upper limit of the hazard ratio (upper confidence interval). ln(HRlci) is the natural logarithm of the lower limit of the hazard ratio (lower confidence interval). z0.975 is the 97.5th percentile of the standard normal distribution, typically 1.96, because the 95% confidence level leaves 2.5% in each tail).


$$se(log(HR))=\frac{ln(HRuci)-ln(HRlci)}{2\times z0.975}$$

Heterogeneity was assessed with I^2^; values <50% indicated low heterogeneity and justified a fixed-effect model, while values ≥50% led to a random-effects model.

Bayesian estimation used four parallel Markov chains, a binomial likelihood, a complementary log-log link, and non-informative normal priors (mean 0, variance 10,000) on treatment effect parameters. After an adaptation phase of 20,000 iterations, 50,000 further samples were drawn to construct posterior distributions and to evaluate density convergence. Convergence was judged adequate when the Gelman-Rubin statistic (R-hat) was <1.05 and trace plots showed adequate mixing. Leave-one-out sensitivity analyses were performed for the primary OS analysis, and frequentist netmeta estimates were compared with Bayesian gemtc estimates as a model sensitivity check. Formal publication-bias testing was not performed because fewer than ten trials contributed to each outcome, making standard tests unreliable.

Results are presented in forest plots and rankograms that show the posterior mean hazard ratio and its 95% Bayesian credible interval (CrI) for each treatment. All analyses were run in R version 3.4.1: the gemtc package fitted the Bayesian models, the coda package summarized the MCMC output, and the netmeta package provided the frequentist checks.

## 3 Results

### 3.1 Quality appraisal

Ten RCTs with a total of 3959 patients were included. The included studies were considered to be of high quality by both assessors (**Figure 2**). A few trials had moderate risk of bias because of incomplete reporting of attrition or outcome data; details are given in (**Supplementary Table S1**). Baseline characteristics were well balanced across studies (**Supplementary Table S2**). All trials were second- or third-line studies, and the distributions of age, sex and prior therapy were similar, supporting transitivity. Because the network was star-shaped and lacked closed loops among active treatments, formal inconsistency testing was not possible; we therefore assessed transitivity by comparing trial characteristics and potential effect modifiers. KRAS prevalence differed between Asian studies (CONCUR 34%, TERRA 37%) and the remaining trials (40-70%); we therefore performed a pre-planned subgroup analysis to account for this imbalance.

**Figure 2:**
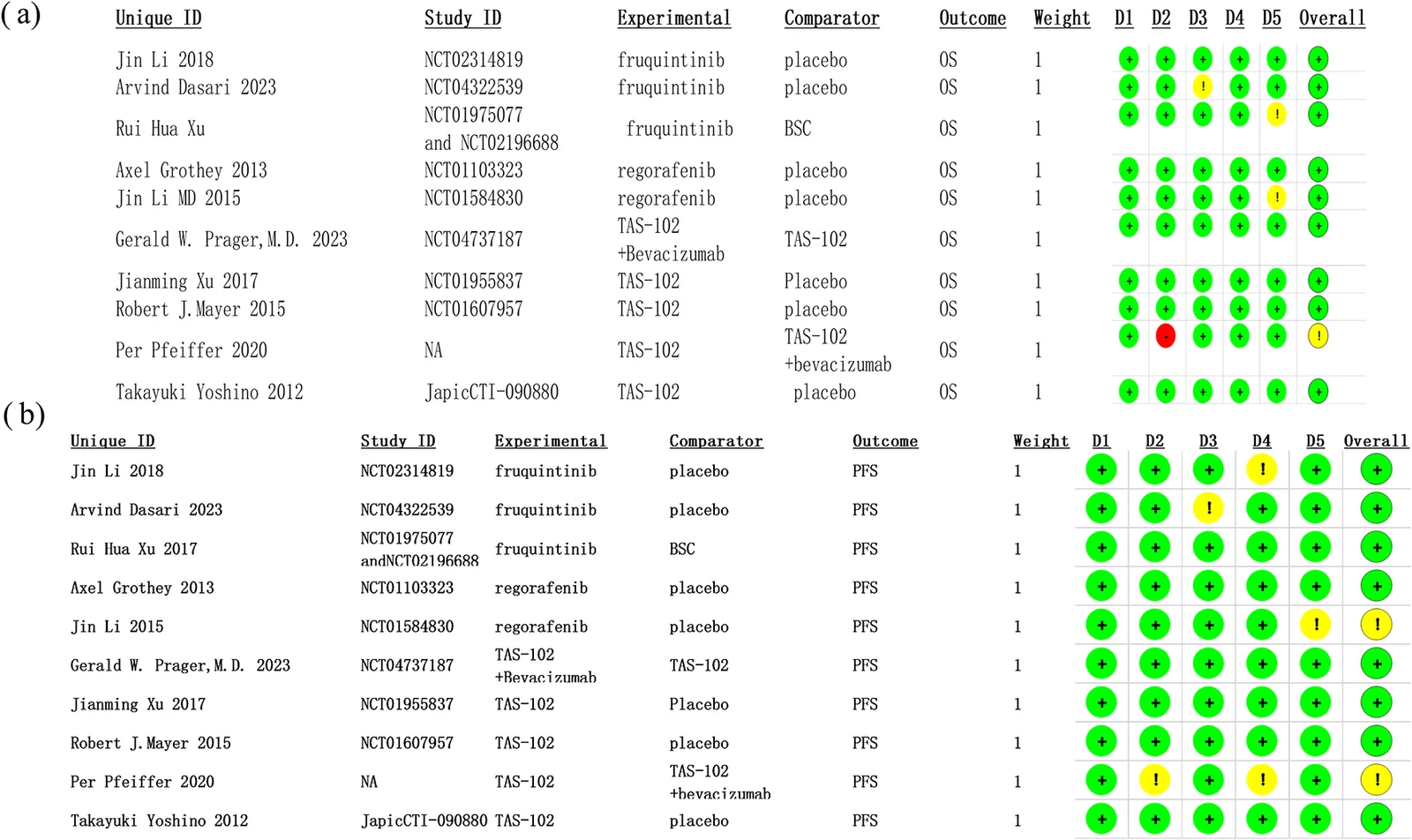
Risk-of-bias analysis. Red indicates high risk of bias, yellow indicates some concerns, and green indicates low risk of bias. D1 represents the randomization process; D2 represents deviations from the intended interventions; D3 represents missing outcome data; D4 represents measurement of the outcome; D5 represents selection of the reported result. **(a)** Risk-of-bias summary for the overall survival outcome. **(b)** Risk-of-bias summary for the progression-free survival outcome.

### 3.2 NMA results

After pooling the extracted data, a Bayesian network model was constructed; the network geometry is shown in **Figure 3**. Density plots of the model parameters indicated good convergence and stable iterations, suggesting adequate model fit and reliable estimates (**Supplementary Figure S1**).

**Figure 3:**
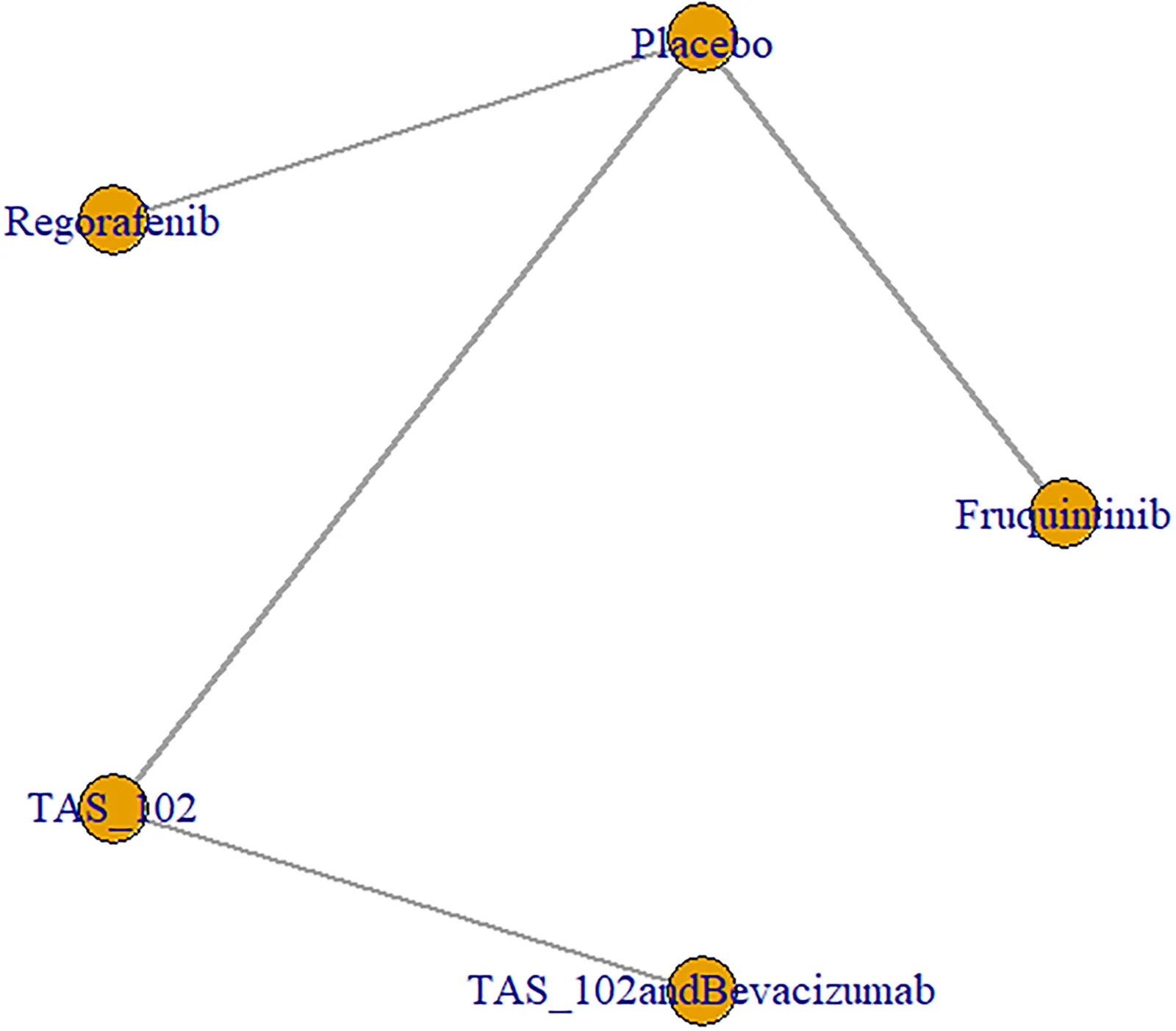
Network geometry.

### 3.3 Analysis of overall survival and progression-free survival

Heterogeneity was low between fruquintinib and placebo (I^2^ ≈ 0%), but was high for regorafenib versus placebo (OS 67.5%, PFS 83.1%) and moderate for TAS-102 versus placebo (OS 21.8%, PFS low; **Figure 4**; **Supplementary Figure S2**). A large part of the observed variability in the regorafenib and TAS-102 trials appears to arise from non-sampling error, accounting for a considerable proportion of the total variance. The excess variation is likely to reflect differences in baseline KRAS prevalence, particularly in the CONCUR and TERRA trials, and led us to use a random-effects model for all pooled estimates.

**Figure 4:**
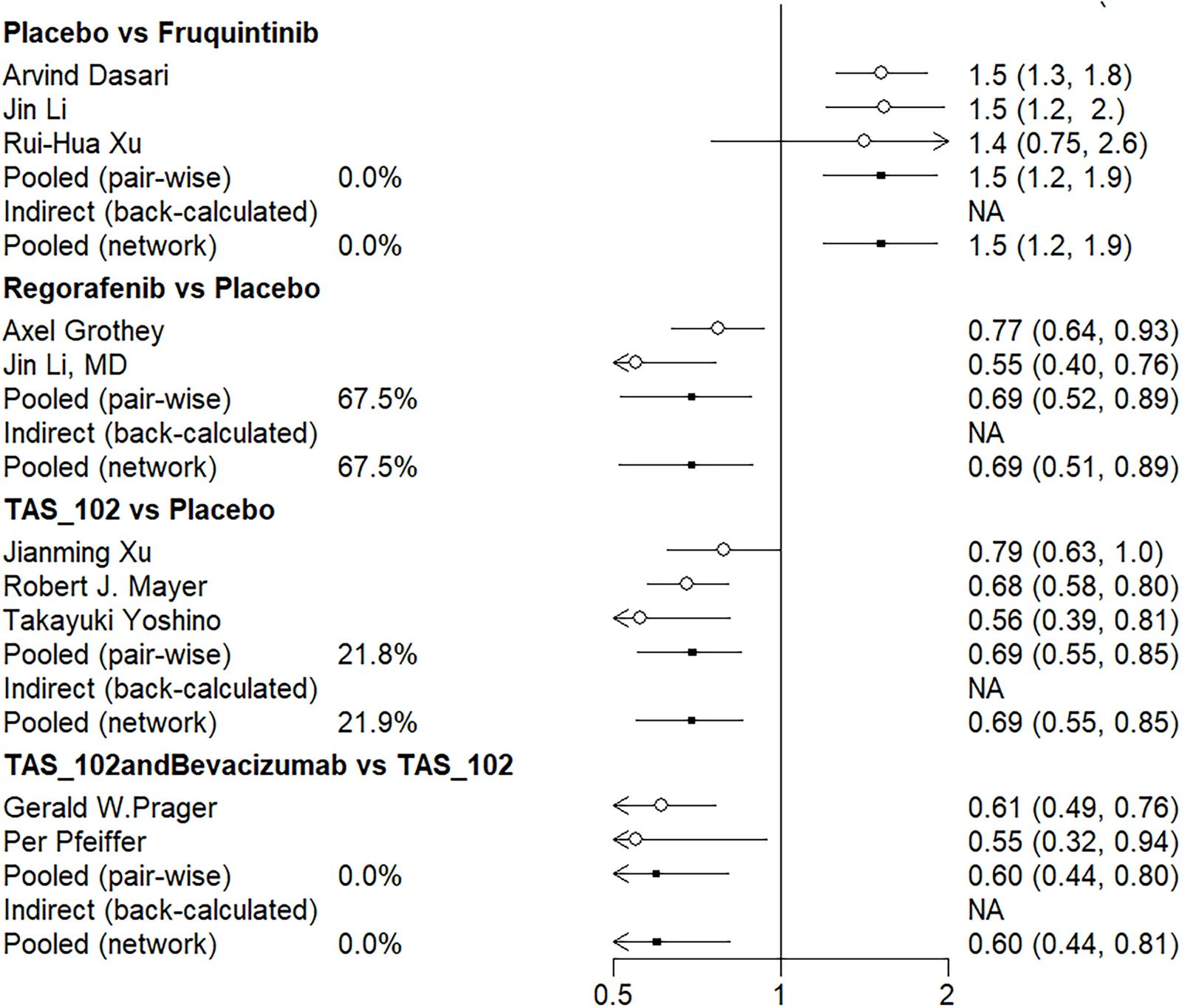
Heterogeneity assessment for overall survival.

Compared with placebo, all active treatments prolonged OS: fruquintinib HR 0.66 (95% CrI 0.53-0.84), regorafenib HR 0.69 (0.51-0.88), TAS-102 HR 0.69 (0.55-0.85), and TAS-102 plus bevacizumab HR 0.41 (0.28-0.59) (**Table 1**; **Figure 5**). Combination therapy ranked first, while the three single agents showed broadly similar efficacy and the indirect differences among them were not statistically significant (SUCRA, **Supplementary Figure S3**). (Dbar 8.86, pD 6.3, DIC 15.17, I2 = 0%) indicated low heterogeneity and reliable estimates.

**Table 1:** Hazard ratios for overall survival by treatment.

	Treatment				
Comparator	Fruquintinib	Placebo	Regorafenib	TAS_102	TAS_102 and Bevacizumab
Fruquintinib	Fruquintinib	1.51 (1.19, 1.9)	1.04 (0.71, 1.45)	1.05 (0.75, 1.42)	0.62 (0.39, 0.95)
Placebo	0.66 (0.53, 0.84)	Placebo	0.69 (0.51, 0.88)	0.69 (0.55, 0.85)	0.41 (0.28, 0.59)
Regorafenib	0.96 (0.69, 1.4)	1.45 (1.13, 1.94)	Regorafenib	1 (0.72, 1.44)	0.6 (0.38, 0.95)
TAS_102	0.96 (0.71, 1.33)	1.45 (1.18, 1.81)	1 (0.7, 1.38)	TAS_102	0.6 (0.44, 0.8)
TAS_102and Bevacizumab	1.6 (1.05, 2.54)	2.43 (1.69, 3.56)	1.68 (1.05, 2.63)	1.68 (1.24, 2.28)	TAS_102andBevacizumab

**Figure 5:**
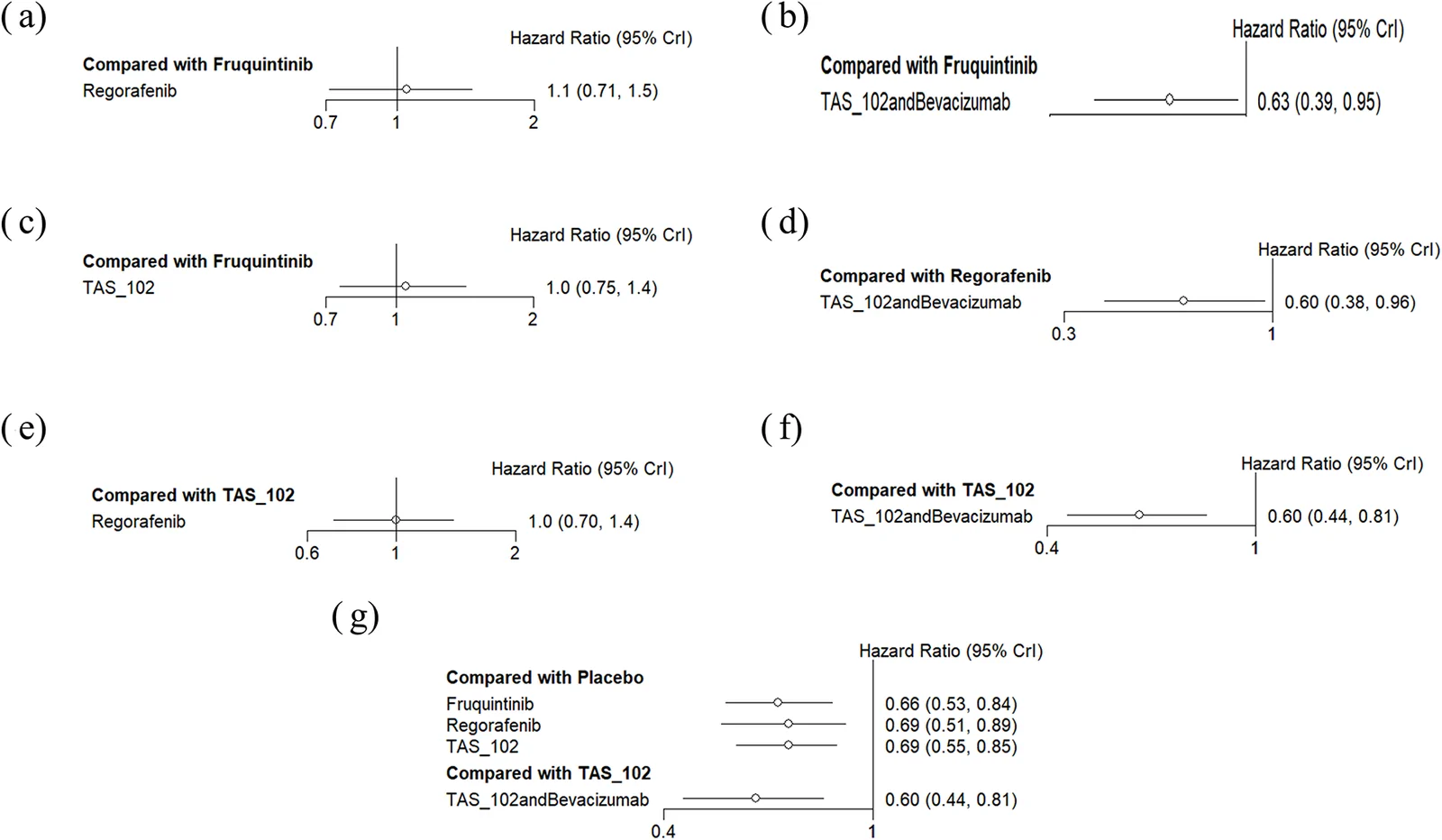
Forest plot of overall survival hazard ratios by drug.

With respect to PFS, all experimental arms outperformed placebo, with the combination again providing the largest benefit (**Figure 6**). In an indirect comparison, fruquintinib showed a more favorable PFS than TAS-102 (HR 0.65; 95% CrI 0.45-0.96) (**Supplementary Table S3**) and ranked highest among monotherapies (**Figure 7**); however, this indirect estimate should be interpreted cautiously in the absence of head-to-head trials. Model diagnostics were satisfactory (Dbar 9.45, pD 7.04, DIC 16.5, I2 = 5%).

**Figure 6:**
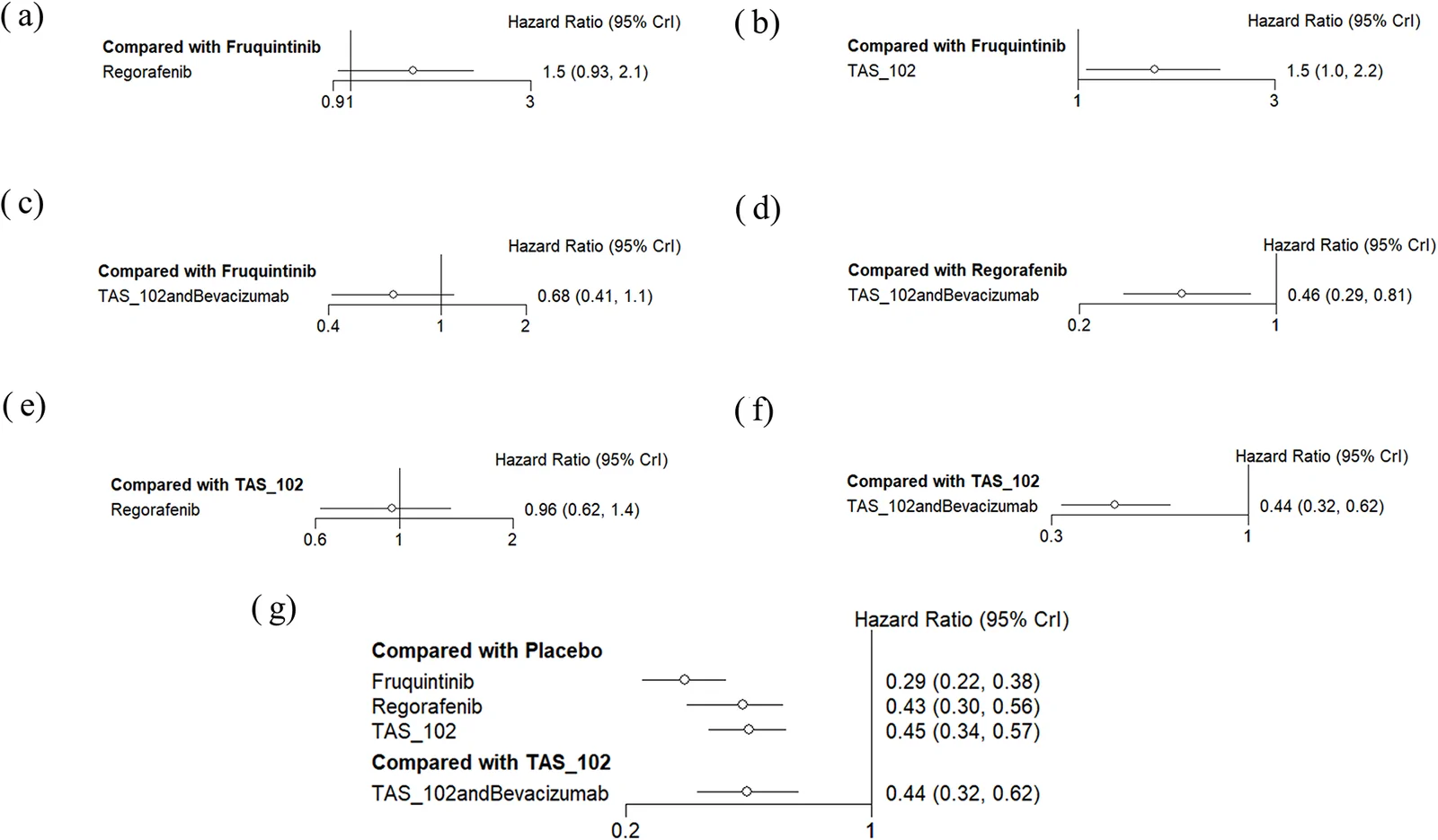
Forest plot of progression-free survival hazard ratios by drug.

**Figure 7:**
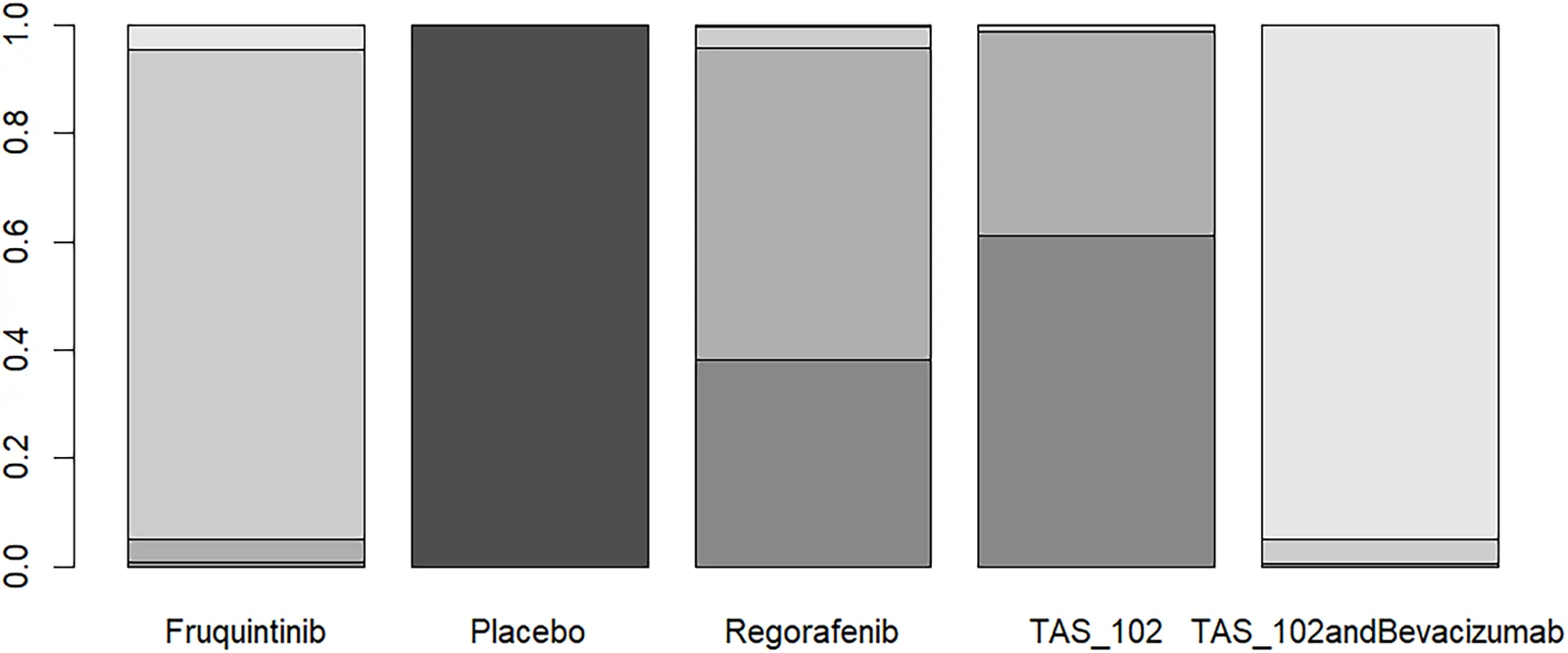
Rank-ordered plot of progression-free survival hazard ratios across treatments.

### 3.4 The analysis of the efficacy of the three drugs in KRAS mutation

Overall, the trials contributing to the regorafenib and TAS-102 nodes showed substantial heterogeneity. In the transitivity checks performed as part of the quality-assessment process, KRAS mutational status emerged as the most plausible source of this heterogeneity. We conducted separate analyses for patients with KRAS-mutant and KRAS-wild-type tumors. Eligibility was restricted to randomized trials that provided an OS hazard ratio for the mutant subgroup, as PFS subgroup data were insufficient for an NMA. The trial by Xu et al. (2017) (36) was excluded because KRAS status was not determined. Individual KRAS variants were not available, and a formal treatment-by-KRAS interaction test could not be performed because individual-participant data were unavailable; consequently, subgroup differences in statistical significance should not be interpreted as evidence of a predictive effect.

#### 3.4.1 Wild-type subgroup

After excluding KRAS-mutant cases, heterogeneity among trials decreased markedly, with I^2^ ≤ 20% for all drug comparison (**Figure 8**). Most between-study variance therefore disappeared, and a random-effects model with similarly low heterogeneity (I^2^ < 20%) was deemed appropriate.

**Figure 8:**
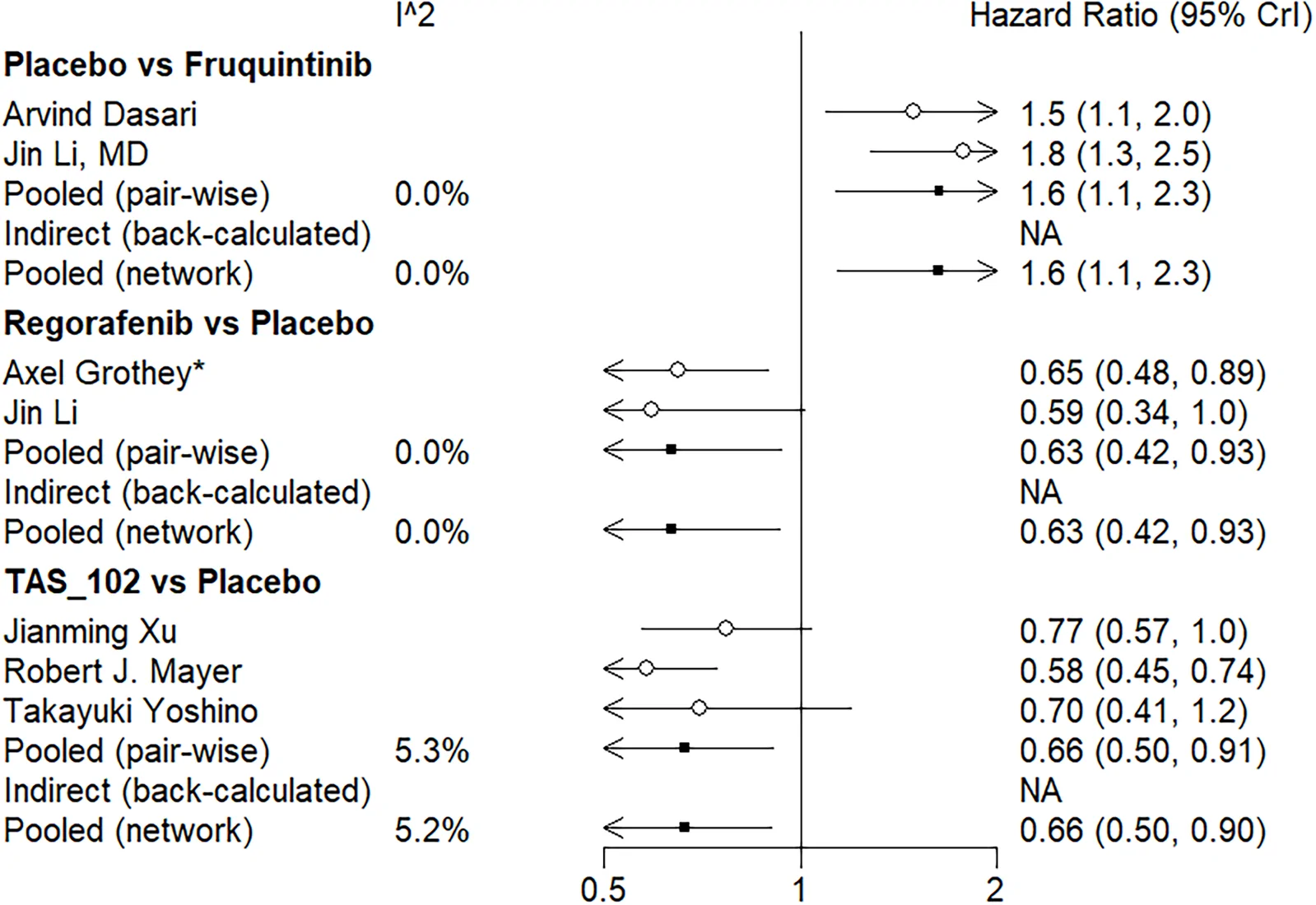
Heterogeneity among RAS-wild-type patients (overall survival).

Forest plots and the league table (**Figure 9**; **Table 2**) show that, when compared with placebo, regorafenib, fruquintinib and TAS-102 each prolonged OS in wild-type patients. However, head-to-head comparisons revealed no meaningful difference between the three agents, and the ranking curve (**Supplementary Figure S4**) positioned them almost side-by-side.

**Figure 9:**
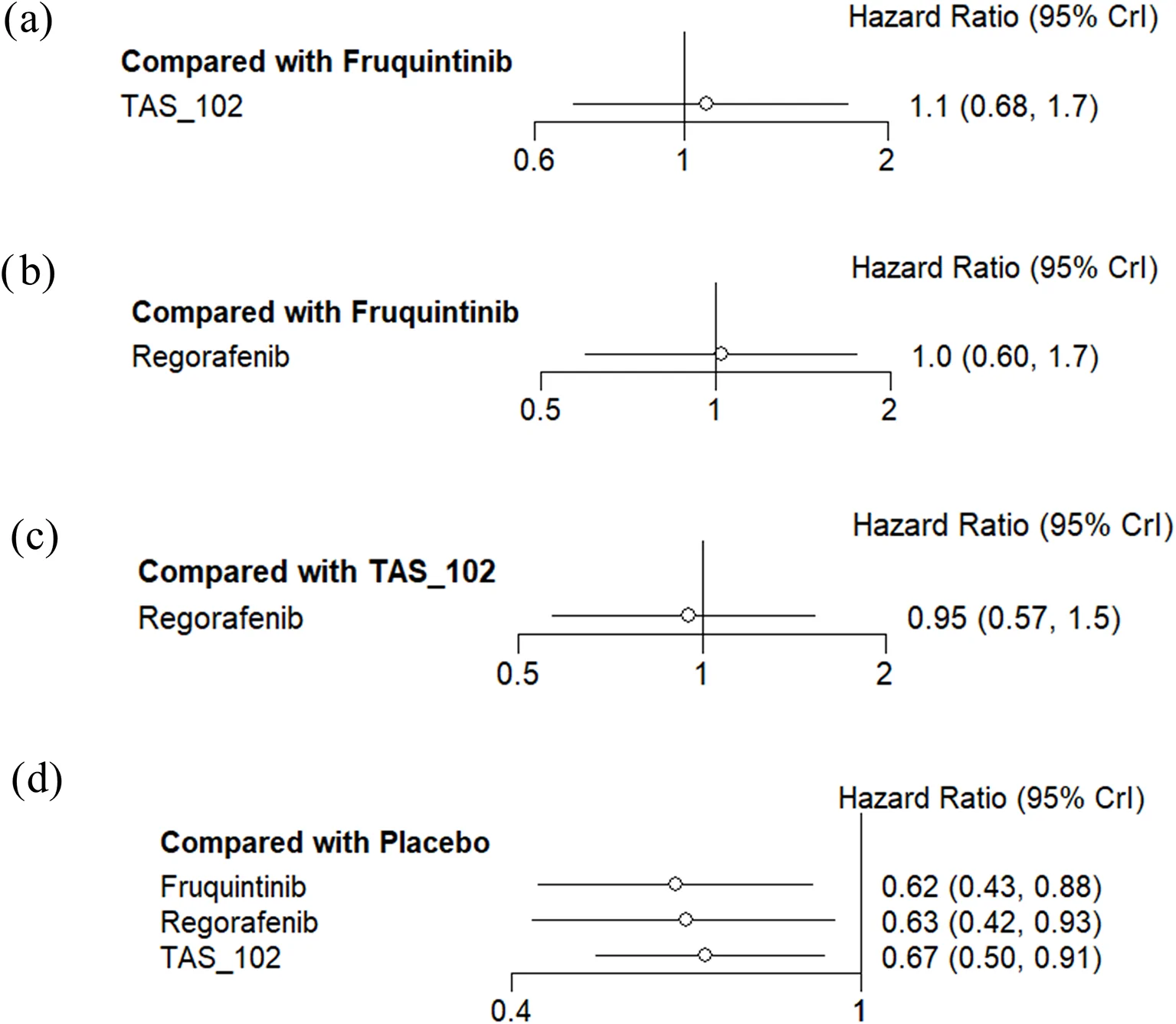
Forest plot of overall survival hazard ratios for each drug in RAS-wild-type patients.

**Table 2:** Hazard ratios for treatment efficacy in KRAS-wild-type patients.

	Treatment			
Comparator	Fruquintinib	Placebo	Regorafenib	TAS_102
Fruquintinib	Fruquintinib	1.62 (1.14, 2.33)	1.02 (0.6, 1.74)	1.08 (0.68, 1.75)
Placebo	0.62 (0.43, 0.88)	Placebo	0.63 (0.42, 0.93)	0.67 (0.5, 0.91)
Regorafenib	0.98 (0.57, 1.67)	1.58 (1.07, 2.37)	Regorafenib	1.05 (0.65, 1.76)
TAS_102	0.93 (0.57, 1.46)	1.5 (1.1, 2.01)	0.95 (0.57, 1.53)	TAS_102

Model fit statistics corroborated these findings: D̄ = 5.56, pD = 4.60, DIC = 10, I^2^ = 0%, indicating virtually no remaining heterogeneity. Thus, in the KRAS-wild-type population the network conclusions closely mirrored those of the overall cohort.

#### 3.4.2 KRAS-mutant subgroup

Heterogeneity was low for fruquintinib and regorafenib (I2 < 20%), but moderate for TAS-102 (I2 = 47.2%), a level still considered acceptable (37). A random-effects model was therefore used (**Figure 10**). Forest plots and the league table show broadly similar OS hazard ratios across the three drugs (**Table 3**). Both fruquintinib and TAS-102 conferred a reduction in the risk of death compared with placebo, whereas regorafenib did not (**Figure 11**). However, these differences in statistical significance versus placebo do not demonstrate that fruquintinib or TAS-102 is superior to regorafenib; the active-treatment comparisons were not statistically significant. The ranking plot places fruquintinib first, followed by TAS-102 and then regorafenib (**Figure 12**), but this ranking is exploratory. Taken together, this subgroup analysis provides a trial-level, KRAS-stratified synthesis of the available placebo-controlled RCT evidence and quantifies the uncertainty in the relative effects of the three agents; it should be interpreted as hypothesis-generating for future prospective, KRAS-stratified or head-to-head studies, rather than as evidence of a predictive effect of KRAS status. Model-fit statistics (Dbar = 7.13, pD = 5.00, DIC = 12.14, I2 = 16%) confirm low heterogeneity and support the reliability of these findings.

**Figure 10:**
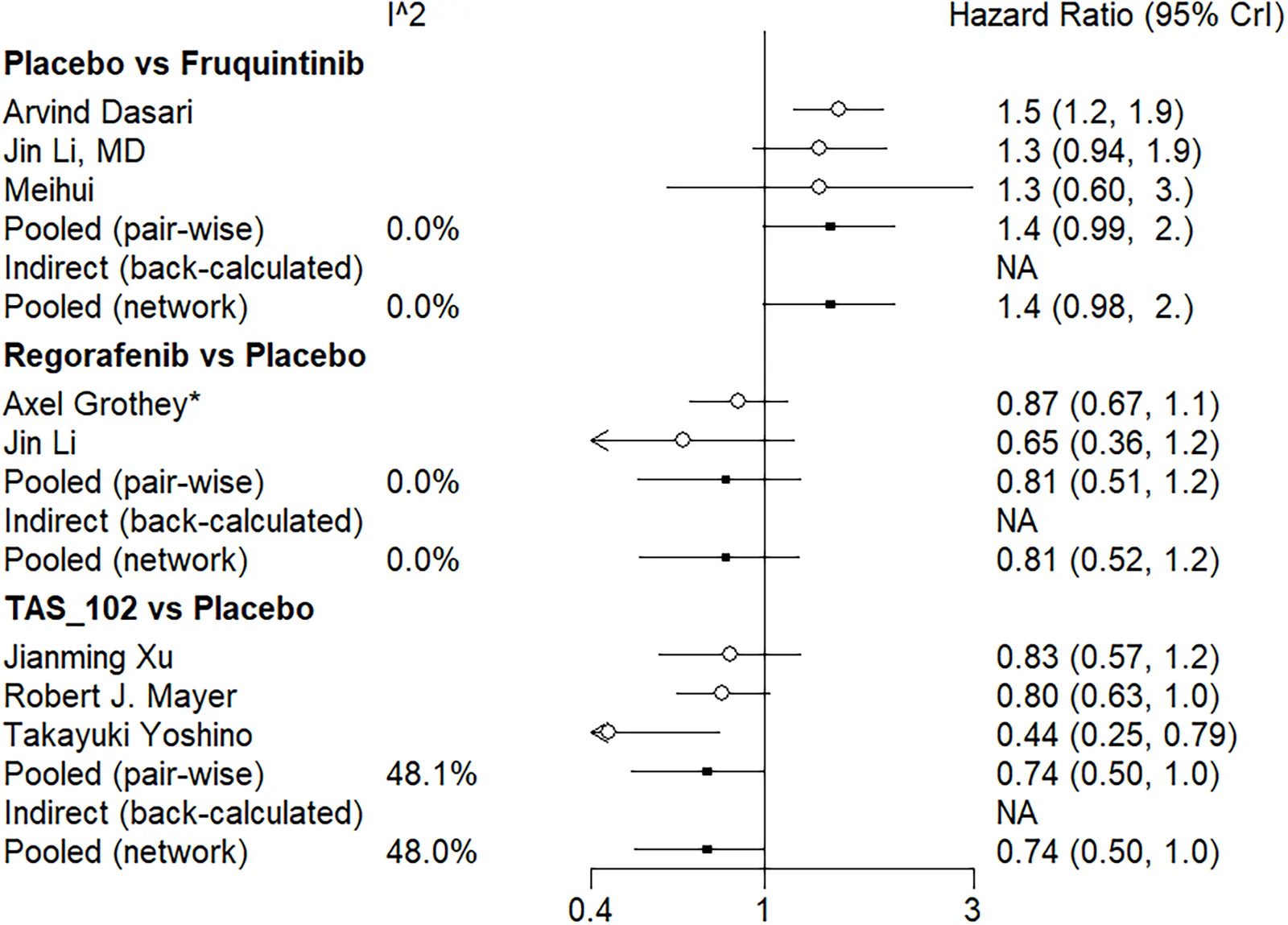
Heterogeneity in KRAS-Mutant Patients (Overall Survival).

**Table 3:** Efficacy of mutant-type drugs on overall survival – hazard ratios.

	Treatment			
Comparator	Fruquintinib	Placebo	Regorafenib	TAS_102
Fruquintinib	Fruquintinib	1.41 (0.99, 1.96)	1.14 (0.64, 1.87)	1.04 (0.61, 1.6)
Placebo	0.71 (0.51, 1.01)	Placebo	0.81 (0.52, 1.19)	0.74 (0.5, 0.99)
Regorafenib	0.88 (0.53, 1.57)	1.23 (0.84, 1.94)	Regorafenib	0.91 (0.53, 1.54)
TAS_102	0.96 (0.63, 1.65)	1.36 (1.01, 2)	1.1 (0.65, 1.9)	TAS_102

**Figure 11:**
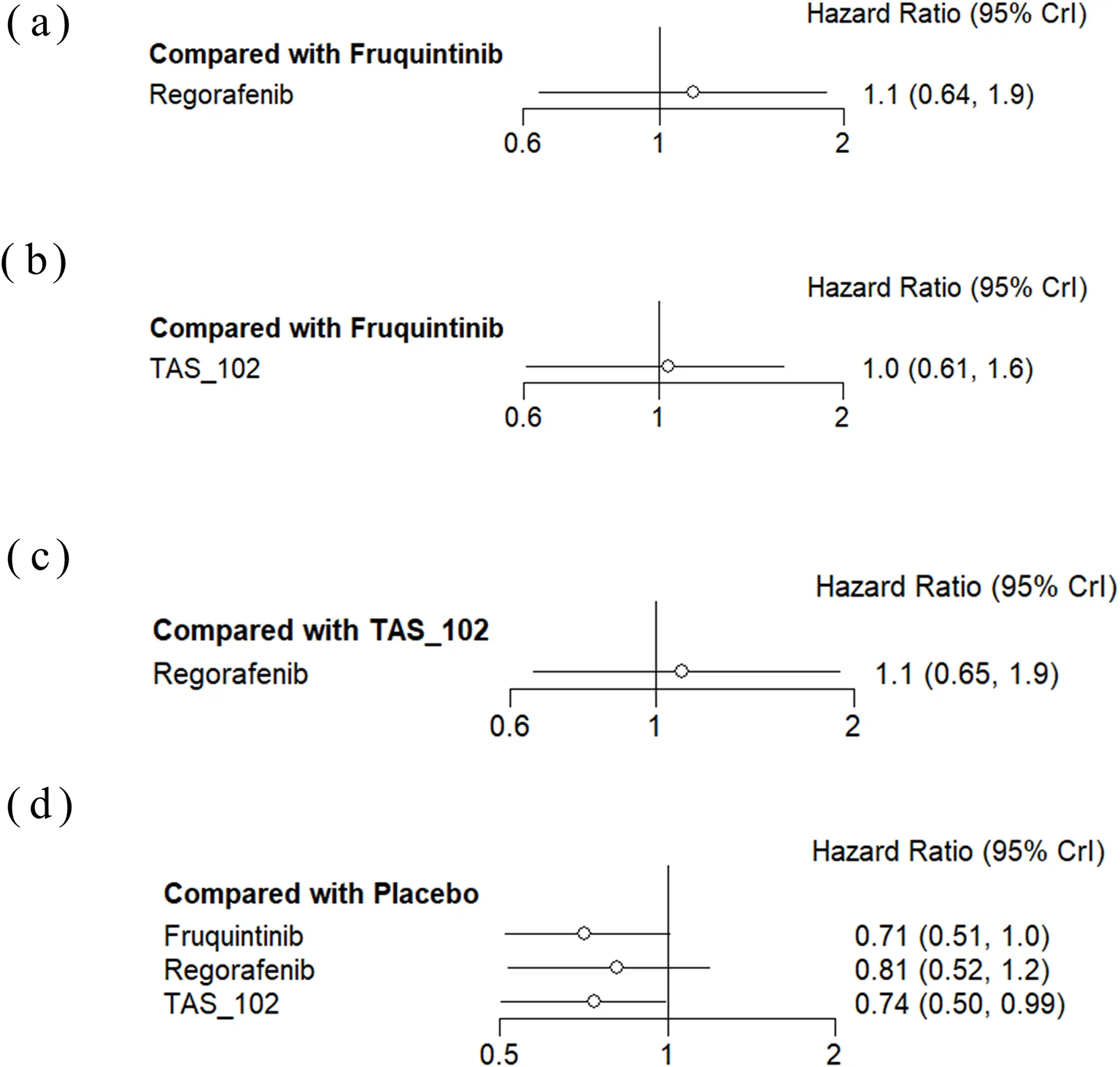
Forest plot of overall survival hazard ratios by drug in KRAS-mutant patients.

**Figure 12:**
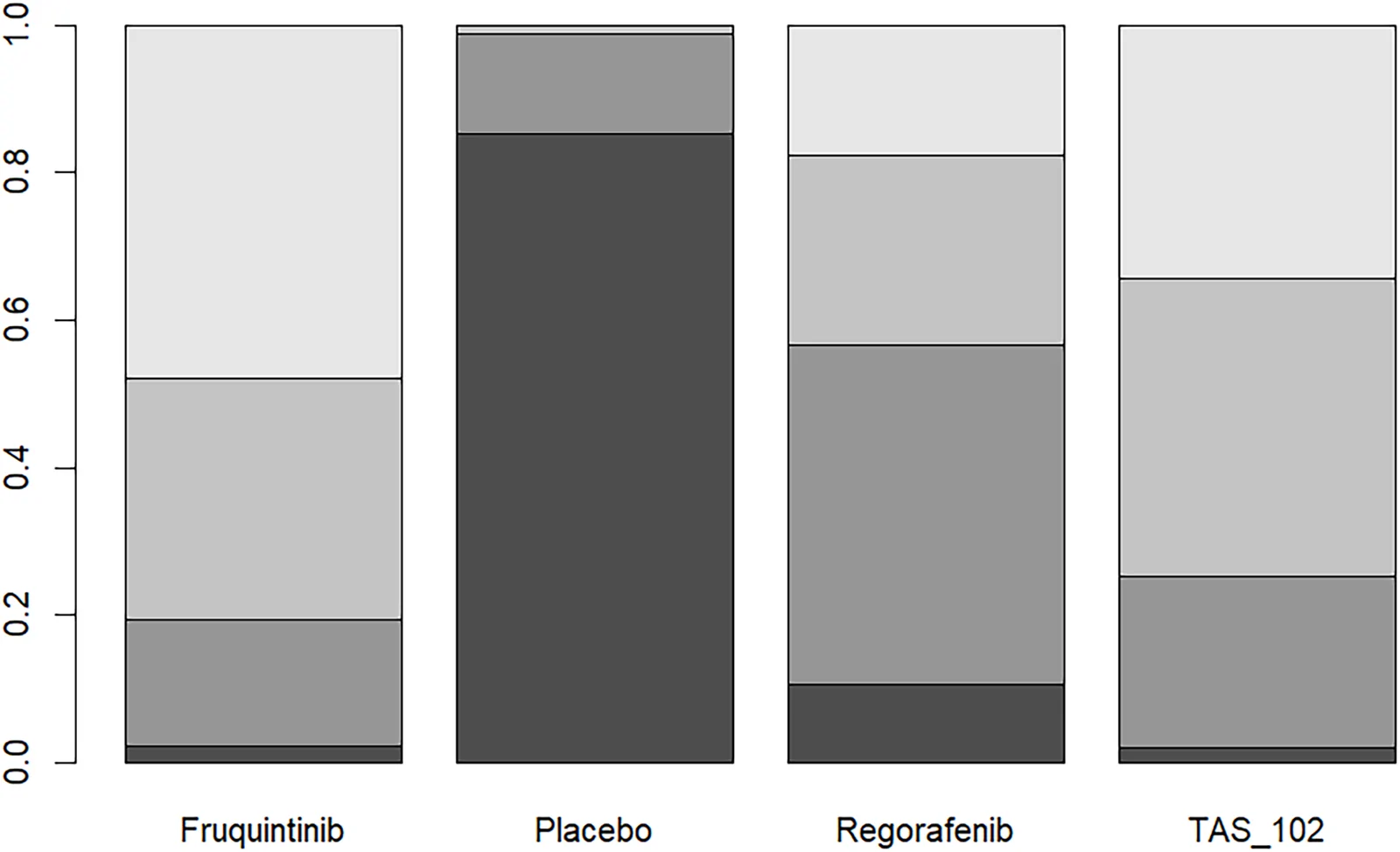
Treatment ranking plot for RAS-mutant patients.

Our network analysis shows that regorafenib, fruquintinib, and TAS-102 all clearly prolong OS compared with placebo. Combining either fruquintinib or TAS-102 with bevacizumab also gives a meaningful PFS benefit. In RAS-mutant patients, rank probabilities suggest an exploratory ordering of fruquintinib, followed by TAS-102 and then regorafenib; this ordering does not establish superiority among the three agents. The contribution of this RCT-based NMA is therefore to quantify the survival benefits of the available later-line options and to provide hypothesis-generating rankings, rather than to establish rank-based superiority.

## 4 Discussion

The network meta-analysis confirmed that fruquintinib, regorafenib and TAS-102 all lengthen OS relative to placebo, while their long-term benefits are generally comparable (28, 38). Combining TAS-102 with bevacizumab was superior to either agent alone, a finding that aligns with recent reports (39, 40). Fruquintinib and regorafenib are both VEGFR inhibitors, yet their pharmacological profiles differ. Fruquintinib is highly selective and has few off-target toxicities, so it can be given at full exposure without excess side-effects; it also blocks both blood and lymphatic vessel formation (41). Regorafenib is a multi-target tyrosine kinase inhibitor that inhibits not only VEGFR, but also FGFR, PDGFR, KIT, RET, and BRAF. This wide target profile enables its use across multiple solid tumors, such as hepatocellular carcinoma, gastrointestinal stromal tumors, and gastric cancer, thereby offering a wider scope of therapeutic indications (28). TAS-102 is characterized by reversible toxicity that can be readily managed through dose modification, resulting in good patient adherence. Moreover, its combination with bevacizumab affords an additional survival benefit (42). Each drug therefore has distinct advantages in mCRC.

When we first compared regorafenib with placebo, heterogeneity was high (OS I^2^ = 67.5%, PFS I^2^ = 83.1%), whereas TAS-102 showed moderate heterogeneity for OS (I^2^ = 21.8%). Baseline checks revealed wide variation in the proportion of KRAS-mutant patients across trials. After stratifying by KRAS status, heterogeneity for regorafenib decreased markedly; for TAS-102 it remained acceptable in the mutant subgroup, so a random-effects model was retained. The final global estimate had I^2^ < 20%, suggesting the summary values are close to the true effects. Among the three TAS-102 trials, the Japanese study by Yoshino et al. demonstrated a more pronounced benefit in patients with KRAS-mutant tumors (HR 0.44) compared with those with wild-type disease (HR 0.70) (43), whereas the remaining two trials reported no difference according to KRAS status (33, 34). This pattern largely explains the modest residual heterogeneity.

Fruquintinib demonstrated the most pronounced PFS advantage, which may be associated with its “tail-effect”: many patients achieved ≥ 6 months of stable or partial disease and remained progression-free well after therapy had ceased (32). Blockade of VEGFR-2/3, normalization of residual vessels and enhanced immune surveillance may represent potential mechanisms underlying this prolonged control. The combination of TAS-102 and bevacizumab provided the most favorable OS and PFS results. In another third-line studies this doublet achieved an HR of 0.41 and was ranked first by SUCRA. Based on existing mechanistic studies, TAS-102 inhibits DNA synthesis and is protected from degradation, while bevacizumab suppresses angiogenesis and remodels the tumor micro-environment; these mechanisms may act synergistically to yield greater effectiveness than either agent alone (44), although prospective studies are required to validate this hypothesis.

In addition to the analyses in previous reviews, we examined KRAS-mutant disease separately, acknowledging the exploratory nature of subgroup analyses and their inherent limitations. KRAS is mutated in approximately 40% of CRC, leading to constitutive RAS signaling and continuous growth (45, 46). Serdar Ata, MD. indicated that treatment response varies by RAS status rather than by primary tumor site (47). Within this population, the exploratory ranking placed fruquintinib first for OS benefit, followed by TAS-102, with regorafenib last; this ranking does not establish clinical superiority. The contribution of this subgroup analysis is therefore to provide a trial-level, KRAS-stratified synthesis of the available RCT evidence and to generate hypotheses for future prospective studies, rather than to establish a predictive effect of KRAS status or superiority of any single agent.

The limited benefit of regorafenib in RAS-mutant tumors has been reported previously. Camaj et al. indicated that exon-2 mutations (especially G12C/A) maintain elevated BRAF-ERK-ELK phosphorylation and attenuate regorafenib-induced apoptosis and vessel shutdown (48). Ye recently identified a KRAS/ERK2–TRIM21–c-Myc/ENO2 glycolytic axis that promotes regorafenib resistance; inhibition of TRIM21-mediated c-Myc degradation keeps elevated glycolysis and diminishes regorafenib efficacy. These data suggest that patients with KRAS mutations may develop resistance to regorafenib (49).

The following supportive data come from observational, single-arm, preclinical or immunotherapy-combination studies and should be regarded as hypothesis-generating rather than as evidence from the randomized-trial NMA. In mCRC studies evaluating combinations of regorafenib or fruquintinib with PD-1/PD-L1 blockade, exploratory evidence from single-arm and real-world studies suggests that fruquintinib-based combinations may have greater antitumor activity. In indirect comparisons, fruquintinib plus immune-checkpoint blockade achieved an objective response rate of 16% (95% CI 13-21%) versus 3% (1-9%) with the regorafenib combination, a disease-control rate of 79% (72-85%) versus 54% (47-61%), and a median PFS of 5.4 months (4.6-6.2) versus 3.0 months (2.5-3.5) (50). Chen’s systematic review of the three approved agents (based on indirect comparisons) likewise indicated that fruquintinib produced a longer PFS (HR 0.57, 95% CI 0.34-0.95) and higher disease control rate (DCR) (OR 1.80, 95% CI 1.08-3.01) than TAS-102 (28). Real-world evidence from a single-arm study reflecting real-world clinical practice, Deng et al. reported that combining either agent with a PD-1 inhibitor produced better outcomes than using the same drug alone (PFS 5.9 vs 3.0 months; OS 17.5 vs 11.3 months). When the two combinations were compared directly in this observational setting, the fruquintinib-based regimen provided significantly longer PFS (5.9 vs 3.8 months; p = 0.018) and OS (17.5 vs 14.8 months; p = 0.044) than the regorafenib-based regimen. The same study noted that altering the sequence of the two drugs also significantly impacted the OS (51). Fruquintinib is a novel VEGFR inhibitor characterized by lower toxicity and higher selectivity, which may maximize therapeutic efficacy when combined with other agents. Therefore, these exploratory data suggest that, in patients with mCRC receiving PD-1/PD-L1 inhibitor therapy, the addition of fruquintinib may result in greater survival benefits, but this requires confirmation in prospective randomized trials.

It is important to note that our study did not conduct an NMA for PFS in the RAS subgroup. The reasons are as follows: First, insufficient sample size led to unstable effect estimates, as evidenced by the extremely wide 95% CI for PFS in the KRAS wild-type subgroup of the CONCUR trial. Second, investigator-assessed evaluation (rather than blinded independent central review) carried a risk of bias, with I^2^ statistics indicating high heterogeneity for PFS ranging from 77.7% to 92.2%, and potential interaction with molecular subtypes. Third, the difference in mechanisms of action between fruquintinib and regorafenib (multi-target versus selective VEGFR inhibition) may introduce biological heterogeneity. In contrast, OS serves as an objective endpoint with a greater number of events, demonstrates lower heterogeneity across RAS subgroups, yielding more robust and reliable results.

The KRAS subgroup analysis of TAS-102 plus bevacizumab was reported in only one study; as network meta-analysis requires at least two studies to form a node, a stable estimate could not be established for this combination, and RAS subgroup analysis was therefore not performed in the present study. The Pfeiffer study demonstrated that combination therapy provided significantly greater survival benefit compared with TAS-102 monotherapy in patients with RAS mutations (HR 0.38, 95% CrI 0.19–0.79). However, no significant difference was observed between combination therapy and monotherapy in the wild-type population. The authors proposed three mechanisms to explain these findings. First, RAS-mutant tumors often exhibit enhanced angiogenic activity and dependence on VEGF signaling, potentially increasing sensitivity to bevacizumab. Second, RAS mutations may be accompanied by changes in the tumor microenvironment, and bevacizumab-induced vascular normalization may enhance TAS-102 distribution and DNA incorporation in tumor tissue. Third, patients with wild-type RAS may have received more prior anti-EGFR therapy, resulting in different response patterns to subsequent treatments (although the proportion of prior anti-EGFR therapy was similar between groups in this study: approximately 40%). These results indicate that combination therapy may provide a new therapeutic approach for patients with KRAS mutations.

## 5 Limitations and future directions

In the comparison of fruquintinib, regorafenib, and TAS-102 for KRAS-mutated mCRC, interaction testing was not performed owing to the lack of raw data. The relatively small number of efficacy studies in patients with RAS mutations may be the primary reason for this phenomenon. In the included RCTs, no head-to-head comparisons of these agents have been conducted in randomized trials, and the star-shaped network precluded formal inconsistency testing. Although P-values could not be calculated due to data limitations, we observed differences in drug rankings across subgroups, which should be regarded only as hypothesis-generating for clinical practice. Differences in statistical significance versus placebo between treatments or subgroups should not be interpreted as evidence of a predictive effect of KRAS status. The contribution of the present analysis is therefore to provide a trial-level, KRAS-stratified synthesis of the available placebo-controlled RCT evidence and to quantify uncertainty in the relative effects of the three agents, rather than to establish a predictive effect of KRAS status or rank-based superiority among treatments. These findings should be viewed as hypothesis-generating and used to inform the design of future prospective, KRAS-stratified or head-to-head trials. This exploratory assumption requires future specialized research to be verified.

Moreover, for these patients with KRAS mutations, outcome measures included only OS, whereas data on secondary endpoints such as ORR, DCR, and duration of response (DoR) were not specifically reported. Individual KRAS variants were not described, and comparative safety was not formally meta-analyzed. Recent work suggests that regorafenib is more active against the G12D RAS variant than against other RAS mutations (52, 53). Furthermore, the number of studies included in the network meta-analysis remains modest, which may affect the statistical power for certain comparisons and precluded reliable publication-bias testing. These limitations suggest that the current findings should be validated by future prospective studies and enriched with real-world evidence.

In conclusion, KRAS mutation testing provides guidance for drug selection and precision medicine in patients receiving second- or third-line therapy. Based on the findings of this study, for CRC patients with wild-type KRAS, standard third-line regimens or the combination of TAS-102 and bevacizumab may be used. For CRC patients with KRAS mutations, fruquintinib may represent a reasonable later-line treatment option; however, the available indirect evidence does not establish its superiority over TAS-102 or regorafenib. The optimal therapeutic choice for these patients should be determined by a comprehensive evaluation that integrates efficacy measures (including PFS, OS, and ORR) with individual patient characteristics, toxicity profiles, and quality-of-life considerations. Overall, this analysis provides a trial-level, KRAS-stratified synthesis of the available placebo-controlled RCT evidence; its findings should be used to inform hypothesis generation and future prospective, KRAS-stratified or head-to-head trials, rather than to claim a predictive effect of KRAS status or rank-based superiority.

## Data Availability

The raw data supporting the conclusions of this article will be made available by the authors, without undue reservation.
