# Supplementary material for: Comparative efficacy of fruquintinib, regorafenib or TAS-102 in patients with KRAS-mutant metastatic colorectal cancer: a systematic review and network meta-analysis
Source: Front Oncol. 2026 Oct 16;16:1948508. doi: 10.3389/fonc.2026.1948508 (PMC13719336; doi:10.3389/fonc.2026.1948508)
Supplement: Supplementary file 1 [file SupplementaryFile1.docx]

**Supplementary Materials**

**Comparative Efficacy of Fruquintinib, Regorafenib or TAS-102 in Patients with KRAS-Mutant Metastatic Colorectal Cancer: A Systematic Review and Network Meta-Analysis**

Yingyu Zhou^1#^；Shiqing Zhao^2#^; Xiaoyi Zheng^2^; Yixuan Wang^2^; Mingzhen Wang^2^; Siyu Chen^2,3^; Zhenan Wu^1^; Dong Zang^1^; Wenzhe Si^2^;

1. Department of Laboratory Medicine, Beijing Hospital of Integrated Traditional Chinese and Western Medicine, Hospital of Integrated Traditional Chinese and Western Medicine, Beijing University of Chinese Medicine, Beijing 100039, China;

2. Department of Laboratory Medicine, Key Laboratory of Cardiovascular Molecular Biology and Regulatory Peptides of National Health Commission, State Key Laboratory of Vascular Homeostasis and Remodeling, Peking University Third Hospital, Beijing 100191, China;

3. Department of Biology, the College of Life Sciences and Health, Wuhan University of Science and Technology, Hubei 430065, China;

Corresponding authors: Professor Zhenan Wu, Professor Zang Dong, MD or Professor Wenzhe Si, PhD, E-mail: [wuzhenan3011@163.com or](mailto:wuzhenan3011@163.com%20or) [zangdmail@163.com](mailto:zangdmail@163.com) or wenzhesi@bjmu.edu.cn (W.Si).

This file includes: Table S1-S3; Figure S1-S4

| **Supplementary Table S1 Experimental Protocols of Included Studies** | | | | | |
| --- | --- | --- | --- | --- | --- |
| **Study** | **Trial** | **Population** | **Experimental Group** | **Control Group** | **Cycle** |
| **Axel Grothey 2013** | CORRECT (Grothey 2013) | Global multicenter (760 patients, including 111 Asians) | Regorafenib 160 mg orally, days 1–21 of a 28-day cycle | Placebo | 28 days |
| **Jin Li, MD 2015** | CONCUR (Jin Li 2015) | Asian population only (204 patients from China, Hong Kong, Taiwan, South Korea, Vietnam) | Regorafenib 160 mg orally, days 1–21 of a 28-day cycle | Placebo | 28 days |
| **Takayuki Yoshino 2012** | Yoshino 2012 | Japan (170 patients) | TAS-102 35 mg/m² orally, BID, days 1–5 and 8–12 of a 28-day cycle | Placebo | 28 days |
| **Jianming Xu 2017** | TERRA 2017 | Asian multicenter (406 patients: 25 centers in China, 3 in South Korea, 2 in Thailand) | TAS-102 35 mg/m² orally, BID, days 1–5 and 8–12 of a 28-day cycle | Placebo | 28 days |
| **Robert J. Mayer 2015** | RECOURSE 2015 | Multicenter in Japan, USA, Europe, Australia (534 patients) | TAS-102 35 mg/m² orally, BID, days 1–5 and 8–12 of a 28-day cycle | Placebo | 28 days |
| **Gerald W. Prager 2023** | SUNLIGHT 2023 | 13 countries, 87 centers in North America, EU, and other regions (246 patients) | TAS-102 + Bevacizumab 35 mg/m² orally, BID, days 1–5 and 8–12 of a 28-day cycle | TAS-102 monotherapy | 28 days |
| **Per Pfeiffer 2020** | Pfeiffer 2020 | 4 oncology centers in Denmark (Odense, Aalborg, South Jutland, Copenhagen) (93 patients) | TAS-102 + Bevacizumab 35 mg/m² orally, BID, days 1–5 and 8–12 of a 28-day cycle | TAS-102 monotherapy | 28 days |
| **Jin Li 2018** | FRESCO 2018 | 28 hospitals in China (519 patients) | Fruquintinib 5 mg orally, QD, days 1–21 of a 28-day cycle | Placebo | 28 days |
| **Arvind Dasari 2023** | FRESCO-2 2023 | 124 hospitals and cancer centers in 14 countries (934 patients) | Fruquintinib 5 mg orally, QD, days 1–21 of a 28-day cycle | Placebo | 28 days |
| **Rui-Hua Xu 2017** | Xu 2017 | China (93 patients) | Fruquintinib 5 mg orally, QD, days 1–21 of a 28-day cycle | Placebo (Phase II) | 28 days |

| **Supplementary Table S2 Patient Characteristics of Included Studies** | | | | | | | | | | |
| --- | --- | --- | --- | --- | --- | --- | --- | --- | --- | --- |
| **Characteristic** | **CORRECT (Global Study)** | **CONCUR (Asian Study)** | **Yoshino 2012 (Japan Phase II)** | **Xu 2017 (Asian Phase III TERRA)** | **Mayer 2015 (Global Phase III RECOURSE)** | **SUNLIGHT 2023** | **Denmark 2020** | **FRESCO-2 (Global Multicenter)** | **FRESCO (China 28 Centers)** | **Xu et al., 2017** |
| **Study Design** | Randomized, double-blind, placebo-controlled, Phase III | Randomized, double-blind, placebo-controlled, Phase III | Randomized, double-blind, placebo-controlled, Phase II | Randomized, double-blind, placebo-controlled, Phase III | Randomized, double-blind, placebo-controlled, Phase III | International, multicenter, open-label, randomized Phase III | Danish 4-center, open-label, randomized Phase II | International, randomized, double-blind, placebo-controlled Phase III | China, randomized, double-blind, placebo-controlled Phase III | Randomized, double-blind, placebo-controlled Phase II (with Phase Ib dose expansion) |
| **Centers/Region** | 16 countries, 114 centers (North America, Western Europe, Israel, Australia, Asia) | 25 centers (China, Hong Kong, Taiwan, South Korea, Vietnam) | 18 centers in Japan | 30 centers (China, South Korea, Thailand) | 114 centers (Japan, USA, Europe, Australia) | 87 centers in 13 countries (64% in EU) | 4 centers in Denmark | 124 centers in 14 countries | 28 hospitals in China | 8 hospitals in China (Phase II), 2 for Phase Ib |
| **Recruitment Period** | 2010-04-30 to 2011-03-22 | 2012-04-29 to 2013-02-06 | 2009-08 to 2010-04 | 2013-10 to 2015-06 | 2012-06 to 2013-10 | 2020-11 to 2022-02 | 2017-08 to 2018-10 | 2020-08-12 to 2021-12-02 | 2014-12 to 2016-05 | April to August 2014 |
| **Randomized (mITT)** | 760 (regorafenib 505 : placebo 255) | 204 (regorafenib 136 : placebo 68) | 169 (TAS-102 112 : 57) | 406 (271 : 135) | 800 (534 : 266) | 492 (246 vs 246) | 93 (47 vs 46) | 691 (Fru 461, Pbo 230) | 416 (Fru 278, Pbo 138) | 71 (Fru 47, Pbo 24) |
| **Age** | Median 61 yrs (IQR 54–67) | Median 57.5 yrs (IQR 50–66) | Median 63 yrs (28–80) | Median 58 yrs (26–81) | Median 63 yrs (27–82) | 62–64 yrs (20–90) | 64–67 yrs (IQR 57–72) | 64 yrs | 54.6 yrs | 54 yrs (IQR 38–70) |
| **Male (%)** | 62% | 63% | 57% | 63% | 61% | 50–55% | 52–64% | 53% | 57% | 70.8% |
| **ECOG 0 / 1** | 52% / 48% | 25% / 75% | 64% / 36% | 24% / 76% | 56% / 44% | 48% / 52% | 50% / 50–68% | 43% / 57% | 28% / 72% | ECOG 0: Fru 12.8%, Pbo 20.8%; ECOG 1: Fru 87.2%, Pbo 79.2% |
| **Primary Tumor Site** | Colon 64% / Rectum 30% / Both 6% | Colon 58% / Rectum 39% / Both 3% | Colon 56% / Rectum 44% | Colon 57% / Rectum 43% | Colon 63% / Rectum 37% | 25–31% | 23–24% | Left 42%, Right 21%, Rectum 31% | Left 77%, Right 20%, Rectum 45% | Colon: Fru 51.1%, Pbo 54.2%; Rectum: Fru 48.9%, Pbo 45.8% |
| **KRAS Mutation** | 54% mutant / 41% wild-type / 5% unknown | 34% mutant / 37% wild-type / 29% unknown | 45% mutant / 55% wild-type | 37% mutant / 63% wild-type | 51% mutant / 49% wild-type | 69% | 59–62% | 63% | 44% | Not tested or reported |
| **Number of Metastatic Organs** | Single organ NR | Single 21% / Multiple 79% | ≥3 organs 39% | ≥3 organs 39% | ≥3 organs 40% | 38–43% | ~40%* | 95% multiple | 95% multiple | Multiple: Fru 95.7%, Pbo 91.7%; Single: Fru 4.3%, Pbo 8.3% |
| **Prior Lines of Therapy** | 1–2 lines 27% / 3 lines 25% / ≥4 lines 49% | 2 lines 23% / 3 lines 24% / ≥4 lines 54% | All ≥2 lines; ≥3 lines 85% | All ≥2 lines; ≥4 lines 50% | All ≥2 lines; ≥4 lines 60% | Only 2–3% | 28–30% | 4th line (73% >3 lines) | 3rd line (~60% ≤3 lines) | 2–3 lines: 60–80% |
| **Median Treatment Duration** | 1.7 mo (reg) vs 1.6 mo (PBO) | 2.4 mo (reg) vs 1.6 mo (PBO) | 1.9 mo | 15 wk (~3.5 mo) | 6.7 wk (~1.5 mo) | 5.0 vs 2.1 mo | 2.4 vs 4.9 mo | 3.1 mo vs 1.8 mo (Pbo) | 3.7 mo vs 1.8 mo (Pbo) | Fru: 3.2 mo; Pbo: 0.8 mo |
| **Dose Modification (reg arm)** | 76% required (20% dose reduction) | 75% required (40% dose reduction) | 85.7% | 98% | 89% | 16% / 69% | 26% / 37% | 24% dose reduction, 47% interruption | 24% dose reduction, 47% interruption | Interruption: 34.0%; Reduction: 27.7% |
| **Primary Endpoint (OS)** | HR=0.77 (0.64–0.94), median 6.4 vs 5.0 mo | HR=0.55 (0.40–0.77), median 8.8 vs 6.3 mo | HR=0.56 (0.39–0.81), median 9.0 vs 6.6 mo | HR=0.79 (0.62–0.99), median 7.8 vs 7.1 mo | HR=0.68 (0.58–0.81), median 7.1 vs 5.3 mo | 10.8 vs 7.5 mo (HR 0.61) | 9.4 vs 6.7 mo (HR 0.55) | 7.4 vs 4.8 mo; HR 0.66 (p<0.0001) | 9.3 vs 6.6 mo; HR 0.65 (p<0.001) | Primary endpoint: PFS |
| **PFS Outcome** | HR=0.49 (0.42–0.58), median 1.9 vs 1.7 mo | HR=0.31 (0.22–0.44), median 3.2 vs 1.7 mo | HR=0.41 (0.28–0.59), median 2.0 vs 1.0 mo | HR=0.43 (0.34–0.54), median 2.0 vs 1.8 mo | HR=0.48 (0.41–0.57), median 2.0 vs 1.7 mo | 5.6 vs 2.4 mo (HR 0.44) | 4.6 vs 2.6 mo (HR 0.45) | 3.7 vs 1.8 mo; HR 0.32 (p<0.0001) | 3.7 vs 1.8 mo; HR 0.26 (p<0.001) | Median PFS: Fru 4.73 mo vs Pbo 0.99 mo (HR=0.30, P<0.001) |
| **Any Grade 3–4 TRAEs** | 54% | 54% | All <1% | All <1% | All <3% | 72% vs 70% | 67% vs 38% | 63% vs 50% (Pbo) | 61% vs 20% (Pbo) |  |

| **Supplementary Table S3 Hazard Ratios for Progression-free survival by Treatment** | | | | | |
| --- | --- | --- | --- | --- | --- |
|  | **Fruquintinib** | **Placebo** | **Regorafenib** | **TAS_102** | **TAS_102andBevacizumab** |
| **Fruquintinib** | Fruquintinib | 3.41 (2.61, 4.5) | 1.46 (0.92, 2.11) | 1.53 (1.05, 2.21) | 0.68 (0.41, 1.11) |
| **Placebo** | 0.29 (0.22, 0.38) | Placebo | 0.43 (0.3, 0.56) | 0.45 (0.34, 0.57) | 0.2 (0.13, 0.3) |
| **Regorafenib** | 0.68 (0.47, 1.08) | 2.33 (1.79, 3.35) | Regorafenib | 1.05 (0.73, 1.62) | 0.46 (0.29, 0.81) |
| **TAS_102** | 0.65 (0.45, 0.96) | 2.22 (1.75, 2.9) | 0.95 (0.62, 1.37) | TAS_102 | 0.44 (0.32, 0.62) |
| **TAS_102andBevacizumab** | 1.48 (0.9, 2.45) | 5.03 (3.34, 7.7) | 2.16 (1.23, 3.49) | 2.26 (1.62, 3.14) | TAS_102andBevacizumab |

**Supplementary Figure S1a Overall Analysis – OS Density Convergence Plot**

**Supplementary Figure S1b Overall Analysis – PFS Density Convergence Plot**

**Supplementary Figure S2 Heterogeneity Analysis of Included Data (PFS)**

**Supplementary Figure S3 Treatment Ranking Plot for Overall Survival by Drug**

**Supplementary Figure S4 Treatment Ranking Plot for Wild-Type Patients**
